# A unique cause of chronic abdominal pain

**DOI:** 10.1002/jgh3.12419

**Published:** 2020-09-16

**Authors:** Yujiro Kawakami, Kotaro Akita, Kazuya Suzuki, Hiroshi Nakase

**Affiliations:** ^1^ Department of Gastroenterology and Hepatology Sapporo Medical University School of Medicine Sapporo Japan; ^2^ Department of Gastroenterology Kushiro City General Hospital Kushiro Japan

**Keywords:** ascariasis, capsule endoscopy, computed tomography, parasite

## Abstract

The images presented here demonstrated how we arrived at the diagnosis of *Ascaris lumbricoides* infection in a 54‐year‐old man with chronic abdominal pain by capsule endoscopy (CE). In this case, computed tomography (CT) images were not representative, and further investigation with CE was required to confirm the diagnosis. The combination of CT and CE was useful for diagnosing Ascaris lumbricoides infection in this patient.

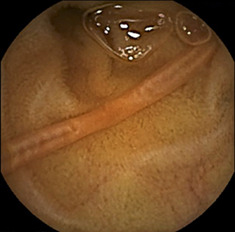

A 54‐year‐old man presented with chronic abdominal pain for 3 months. He had no record of overseas travel, and he was a vegetarian. Blood tests revealed no abnormalities. Esophagogastroduodenoscopy and colonoscopy revealed no significant findings, but contrast‐enhanced computed tomography (CT) revealed bowel wall thickening in the jejunum (Fig. 1a: axial image, arrow; Fig. 1b: coronal image, arrow heads). Capsule endoscopy (CE) was performed to evaluate the small bowel thickening on CT. CE detected a substantial number of intestinal parasites in the jejunum (Fig. 1c,d). Further laboratory investigations revealed elevation of anti‐*Ascaris* immunoglobulin E levels (19.1 UA/mL). He was diagnosed with *Ascaris lumbricoides* infection and received pyrantel pamoate. His abdominal pain symptoms improved immediately after he received it.

**Figure 1:**
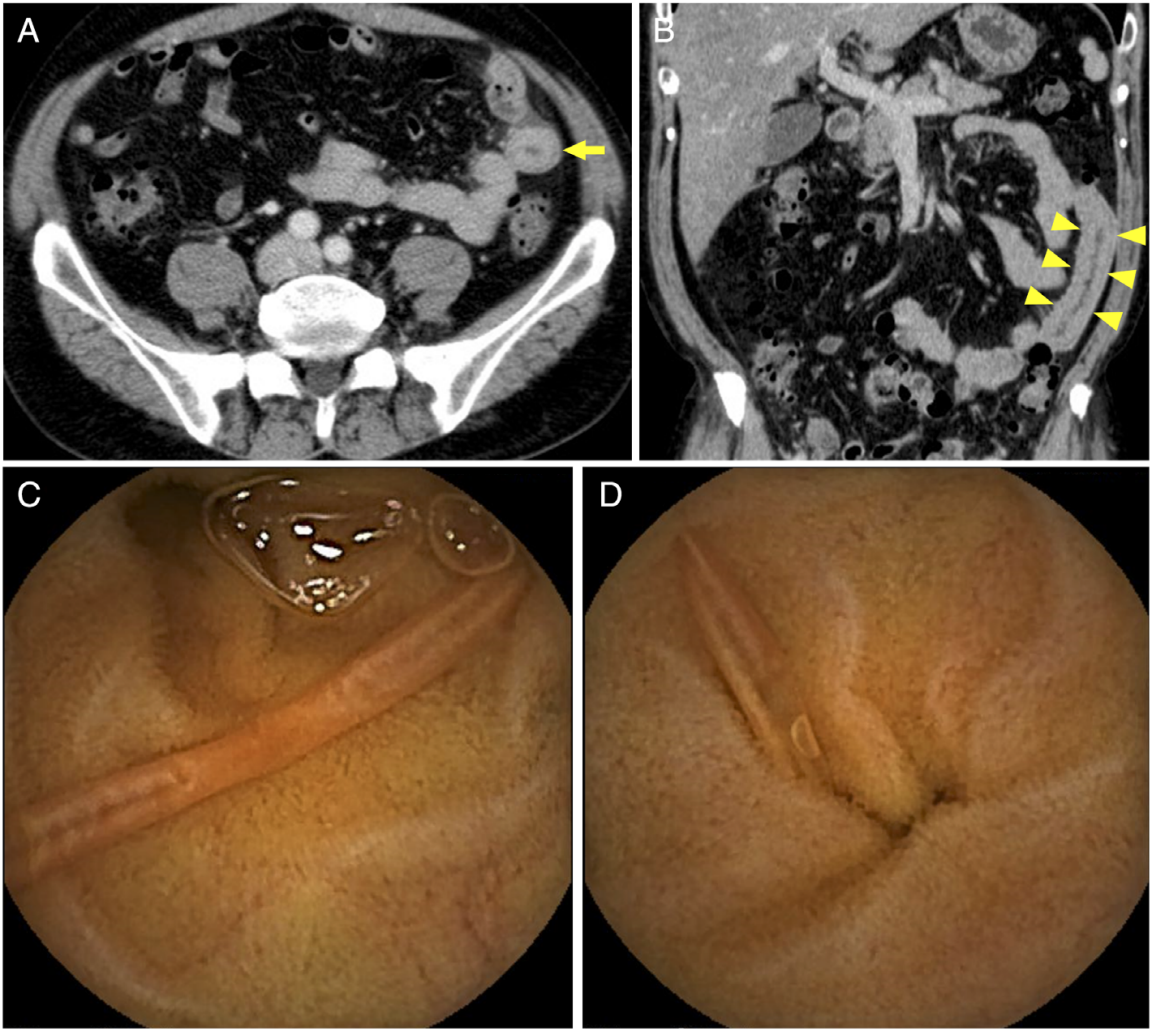
(a) Axial and (b) coronal views of computed tomography showing bowel wall thickening in the jejunum (arrow and arrow heads). (c, d) Capsule endoscopy detected a substantial number of intestinal parasites in the jejunum.

*A, lumbricoides* is an intestinal roundworm parasite that can cause pulmonary, intestinal, pancreatic, or hepatobiliary complications.^1^, ^2^, ^3^ CT imaging of the worm in cross section demonstrates a “bull's eye” appearance.^4^ However, the CT image in this case was not representative, and further investigation with CE was required to confirm the diagnosis. The combination of CT and CE is, therefore, useful for the diagnosis of patients with *A. lumbricoides* infection who complain of chronic abdominal pain.

## Supporting information


**Video S1.** Supporting information.
